# Identifying core components and indicators of successful transitions from child to adult mental health services: a scoping review

**DOI:** 10.1007/s00787-018-1213-1

**Published:** 2018-10-08

**Authors:** Kristin Cleverley, Emily Rowland, Kathryn Bennett, Lianne Jeffs, Dana Gore

**Affiliations:** 1 CAMH Chair in Mental Health Nursing Research, Lawrence S. Bloomberg Faculty of Nursing and Department of Psychiatry, University of Toronto, 155 College Street, Suite 130, Toronto, ON M5T 1P8 Canada; 2 Centre for Addiction and Mental Health, Toronto, Canada; 3 Dalla Lana School of Public Health, Social and Behavioural Health Sciences, University of Toronto, Toronto, ON Canada; 4 Department of Health Research Methods, Evidence and Impact (Formerly Clinical Epidemiology and Biostatistics), McMaster University, Hamilton, Canada; 5 Offord Centre for Child Studies, McMaster University, 1280 Main St W, Hamilton, ON L8S 4L8 Canada; 6 Lawrence S. Bloomberg Faculty of Nursing, University of Toronto, Toronto, Canada; 7 Keenan Research Centre of the Li Ka Shing Knowledge Institute, St. Michael’s Hospital, 30 Bond Street, Room 720, Toronto, ON M5B 1W8 Canada

**Keywords:** Transition, Mental health, Youth, Scoping review

## Abstract

The aim of this scoping review was to identify the core components of interventions that facilitate successful transition from child and adolescent mental health services to adult mental health services. In the absence of rigorous evaluations of transition program effectiveness for transitioning youth with mental health care needs, these core components can contribute to informed decisions about promising program and intervention strategies. This review examined data from 87 peer-reviewed and non-academic documents to determine the characteristics that support the transition process and to identify opportunities for system and program improvement. Data were extracted and synthesized using a descriptive analytic framework. A major finding of this review is a significant lack of measurable indicators in the academic and gray literature. This review did identify 26 core components organized within the framework of the six core elements of healthcare transitions. Policy makers, practitioners, and administrators can use the core components to guide decisions about transition program and intervention content. Confirmation of the impact of these core program components on youth outcomes awaits the conduct of rigorous randomized trials. Future research also needs to explicitly focus on the development of indicators to evaluate transition programs and interventions.

## Introduction

Optimal mental healthcare is crucial throughout the life course, yet the transition from child and adolescent mental health services (CAMHS) to adult mental health services (AMHS) has been recognized as a uniquely problematic international health systems hurdle [1–9]. This common demarcation in mental health services that occurs at age 18, when youth must transition from CAMHS to AMHS, creates the risk of transition-related discontinuity of care. Consequently, these youth are vulnerable to decrements in their mental health and functioning [10–13].

In mental healthcare, transition is distinct from transfer of care. Transfer is the termination of CAMHS and re-establishment of care in AMHS [14]; transition aims to ensure continuity of care through a planned, personalized, health care process that addresses therapeutic and developmental needs [15]. Continuity of care is defined by: (a) how a patient experiences care over time and (b) how care is received by a patient across the episodes of care [16]. Continuity of care must be coherent and linked as a result of good information flow, interpersonal and readiness skills of the youth, and coordination of care [16]. Successful continuity of care is contingent upon optimal transition from CAMHS to AMHS. However, the transition process is often a negative one for youth and their caregivers [17, 18] and as many as six of ten youth stop accessing mental health services [13, 19]. Even with this recognition, there is a paucity of research evaluating interventions that improve the transition process for youth as they move from child to adult services [20].

A recent scoping review of interventions to support CAMHS–AMHS transitions revealed that very few interventions have been developed and evaluated, and that none were standardized [21]. The lack of evaluated programs and interventions may be due in part to a lack of consensus on what the core components and corresponding quality and process indicators of successful transitions are. Core components are defined as the most essential and indispensable components of an intervention or program and should identify how the traits are developed, if they are replicable and the environments in which they can be observed [22]. Indicators are quantitative measures that can be used to monitor and evaluate the quality of interventions and outcomes of CAMHS–AMHS transition programs [23]. This gap in standardized and evaluated interventions has remained unchanged in recent years, even though experts identified understanding “how to measure a successful transition” or “evaluate a transition program” as two key research priorities more than 5 years ago [24].

It is important to address these gaps as not only are clinicians, researchers, and policy demanding greater understanding of the mental healthcare transition process but youth and their families are requesting improvements to continuity of mental health care [1, 3, 25–27]. Hence, this scoping review explores the literature on mental health transitions to identify core components and indicators that facilitate CAMHS–AMHS transition programs and interventions.

## Methods

This scoping review applied the six-stage scoping review framework of Arksey and O’Malley [28, 29] as outlined in the study protocol [30].

### Stage 1: research questions

1. How has a ‘successful transition’ from CAMHS to AMHS been defined and operationalized?
2. What core components and indicators have been used to define and evaluate CAMHS–AMHS transition programs and interventions?

### Stage 2: search strategy

The search strategy was developed by an experienced research librarian. For the published literature, the following databases were included: Medline, Embase, PsycINFO, Cochrane Database of Systematic Reviews (all OVID interface), CINHAL (EBSCO), Applied Social Science Index and Abstracts (ProQuest), Sociological Abstracts (ProQuest) and the Campbell Library. In addition, the gray (unpublished) literature search included the following databases: Google Advanced and the Canadian Agency for Drugs and Technologies in Health search tool, ‘Grey Matters’ [31]. The detailed search strategy is listed in an Appendix in the published protocol [30]. In summary, we used a combination of controlled vocabulary and text words for the key concepts of transition and child, adolescent, and adult mental health. The gray literature search used similar search terms and targeted child/adolescent mental healthcare providers, agencies and support services. The search was limited to English language studies published between 1980 and 2016. The reference lists of relevant articles were searched by hand to identify articles and documents that were not generated in the database search.

### Stage 3: study selection

#### Title and abstract review

Study selection began with a review of the titles and abstracts based on inclusion criteria. All research studies (experimental, quasi-experimental, observational) and non-research studies (guidelines, narrative reviews, policy documents) examining the transition from CAMHS to AMHS were included. Title and abstract review was not completed on the gray literature given the length of these documents and the lack of abstracts. Inclusion criteria consisted of research and non-research publications that examined the transition from CAMHS to AMHS. Studies from acute care, primary care and community services were included. Studies and documents that focused primarily on the developmental disorders, autism, and associated intellectual and developmental disabilities (IDD) were excluded given the primary focus of the review on mental disorders as the diagnosis.

Two researchers (KC and a graduate student) pilot tested the eligibility criteria using a random sample of 25 articles. Using the Cohen *k* coefficient, the inter-observer agreement of the pilot test was kappa 0.88 (95% CI 0.66–1.00), which is considered almost complete agreement [32]. Using Cochrane review software (Covidence), the reviewers then independently screened each retrieved title and abstract for eligibility using the inclusion criteria.

#### Full-text review

The full text of the remaining peer-reviewed articles and all gray literature documents were then screened by KC and a graduate student for eligibility. In the event of a conflict, a third reviewer (ER) was consulted for resolution.

### Stage 4: charting the data

Data were extracted from all eligible articles and entered into Microsoft Excel by the second author (ER). As detailed in the protocol [30], data extracted included: publication type, study aim/objectives, definition of transition, definition of transition success, description of transition intervention or program, description of core components of transition, name and description of indicator, indicator type (process, outcome), methods for measurement, evidence to support the indicator, and results (if applicable). The first author (KC) extracted data from five randomly selected articles to ensure reliability of the content entered in each category.

Data were then organized and analyzed using a directed content analysis approach [33], referring back to the research questions as a guide [29]. Duplicate components or characteristics were removed, and categories with the same meanings collapsed. This iterative process facilitated the identification of how transition was being defined in the field and how larger themes would be determined. Some extracted data fit into more than one category. The research team reviewed the data and categories as a group to achieve consensus. A third reviewer with a Master’s level education was included in the data extraction process to ensure validity and reduce bias.

### Stage 5: collating, summarizing and reporting results

The team organized the content according to the broad categories described in stage four to reflect the patterns and unique explanations. To answer the first research question, definitions of transition success were collated, and then the number of times a particular definition was utilized across documents was recorded. The data extracted on the core components and indicators of CAMHS–AMHS transition (question 2) were summarized using a directed content analysis framework [33]. This entailed reviewing the data and identifying which stage of the transition process it described or informed. To ensure reliability and agreement of the content being extracted, the two researchers (KC and ER) met frequently to review each stage of the analysis and the data captured in each category.

## Results

### Characteristics of documents

**Fig. 1:**
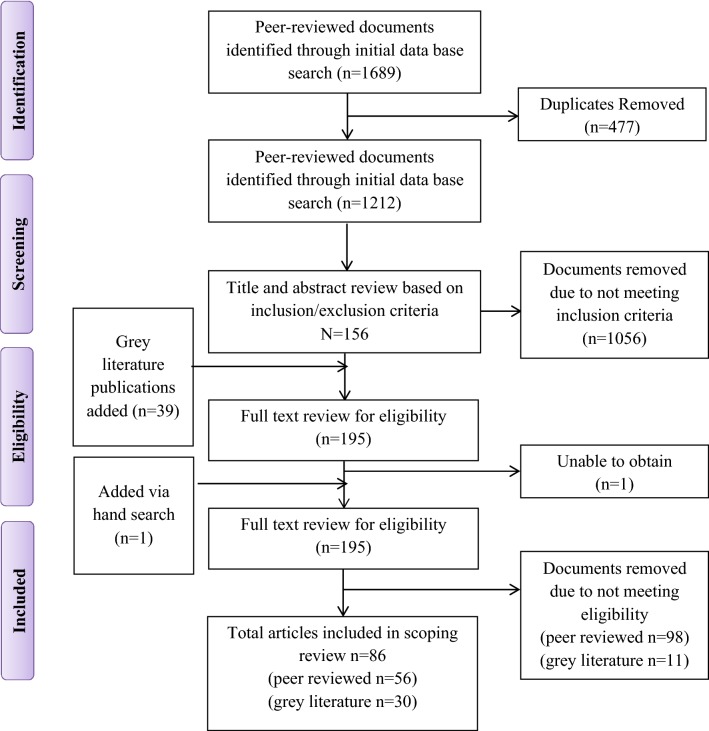
Flow chart of search results

Initial database searches yielded 1212 articles for screening (see flow chart, Fig. 1). Following title and abstract screening, 156 peer-reviewed documents met the study inclusion criteria and were retrieved for full-text review. The 39 documents from the gray literature search were then included for a total of 195 documents for full-text review. After full-text review, 86 documents were eligible: 56 peer-reviewed journal publications and 30 gray literature documents. All articles were published between 1990 and 2016 with the majority (81%) being published since 2010. In total, 55 of the articles (65%) were published studies from the UK, 11 from the US, 9 from Canada, 3 from Italy, 2 from Ireland, 2 from Sweden, 2 from Australia, and 1 from Scotland. The peer-reviewed publications included qualitative studies (*n* = 14), quantitative studies (*n* = 17), and reviews (*n* = 9). The remaining non-original research consisted of discussions, theoretical perspectives (*n* = 8) and commentaries (*n* = 16). The gray literature mostly consisted of program reports (*n* = 11) or guidelines aimed at policymakers and health care providers (*n* = 8). The remainder were briefings (*n* = 1) and position statements (*n* = 2). While most (*n* = 67) papers and documents focused broadly on mental health conditions, few focused on specific mental health diagnosis such as attention deficit/hyperactivity disorders (*n* = 15) and eating disorders (*n* = 4).

### Question 1: operationalization of child to adult mental health transition success

No standard definition of CAMHS–AMHS transition success was applied consistently across the literature, although there was consensus on some aspects. For example, three articles point to transition being a process and not a single event in time [14, 34, 35]. There was consistent use of two definitions of mental health transitions throughout the documents, with 22 papers citing the same two references, one broadly related to health transitions in general [15] and the other specific to CAMHS–AMHS transitions [13]. The first is from Blum et al. [15], who conceptualize a transition as “the purposeful, planned movement of adolescents and young adults with chronic medical conditions from child-centered to adult-oriented health care systems”. Blum’s definition was referred in nine of the reviewed articles, yet it is not specific to transitions in mental health care [3, 7, 13, 14, 36–40].The second is from Singh et al. [13], who build on the definition from Blum et al. [15], adding four specific criteria for determining whether transitions in care were optimal or successful. Further, unlike Blum et al. [15], these criteria are specific to transitions from CAMHS to AMHS. These criteria were used as definitions and key elements for transition success in 15 articles reviewed [5, 11, 13, 14, 20, 39, 41–49]. The criteria include: (a) information transfer: evidence that a referral letter, summary of CAMHS care, or CAMHS case notes were transferred to AMHS along with a contemporaneous risk assessment; (b) period of parallel care: a period of joint working between CAMHS and AMHS during transition; (c) transition planning: at least one meeting involving the service user and/or carer and a key professional from both CAMHS and AMHS prior to transfer of care; and (d) continuity of care: either engaged with AMHS 3 months post-transition or appropriately discharged by AMHS following transition.

### Question 2: core components and indicators

None of the publications reviewed by the research team described quantifiable indicators as defined by Mainz [23]. However, the papers reviewed did describe and recommend components of interventions or programs that promoted successful transitions from child to adult mental health services. One particular framework found in the reviewed literature, Got Transition [12], identified six core elements and corresponding core components of transitions from child to adult physical health services. These six core elements (transition policy, transition tracking and monitoring, transition readiness, transition planning, transfer of care and transfer completion) are designed to guide interventions and programs for child to adult transition in physical health care. The research team used these six core elements as the overarching themes to organize our scoping review’s core component findings [12, 50]. However, the components in this scoping review are specific to CAMHS–AMHS transitions. Table 1 presents the core elements, core components, and corresponding references included in this review.

**Table 1:** List of core elements, components and corresponding references

Core elements	Core components	References
(1.0) Transition policy	(1.1) Develop an integrated care pathway that describes the steps that make up the transition process	[1, 3, 20, 44, 46, 48, 49, 51–53, 57, 59–61, 66, 67, 71, 73, 76, 80, 85–88, 90, 91, 93]
	(1.2) Develop a transition policy/statement with input from youth and caregiver(s) that describes the program/agencies approach to transitions	[1, 3, 13, 35, 46, 52–60]
	(1.3) Develop a needs-led and developmentally appropriate transition protocol that is agreed upon by both CAMHS and AMHS, which includes standards for communication, information sharing and record-keeping	[1, 3–5, 13, 35, 40–42, 44–46, 48, 50–52, 54–60, 62, 63, 65–74, 79–81, 83, 85, 86, 89–91, 93–99, 101]
	(1.4) Ensure that all staff have the knowledge, skills and training to effectively support the agency/services approach to transitions	[1, 3, 4, 13, 20, 34, 35, 38, 39, 42–44, 49, 54, 57, 60–80]
	(1.5) Determine a clear role for all individuals (youth, caregivers, CAMHS and AMHS staff) involved in the transition process	[3, 13, 20, 34, 35, 56, 59, 60, 71, 76, 81–83]
	(1.6) Include young person and their caregiver(s) at all phases of transition and decision-making	[1, 5, 13, 20, 34, 37, 39, 43–45, 49, 51–57, 59, 60, 62, 63, 65, 67, 70, 73, 75, 76, 79, 81–83, 85, 86, 88–91, 98, 99, 101]
	(1.7) Establish the process for evaluation and assessment of transition protocol	[34, 52, 54, 56, 59, 62, 63, 69–71, 75, 76, 79, 90, 98, 103]
(2.0) Transition tracking and monitoring	(2.1) Establish criteria and process for identifying youth who will be transitioning in and out of the agency/service	[26, 52, 54, 63, 64, 76, 78, 80, 85, 103–105]
	(2.2) Establish a transition flow sheet or log book to track youth who transition between CAMHS and AMHS	[50, 54, 61, 76, 104, 106]
(3.0) Transition readiness	(3.1) Conduct regular transition readiness assessments, and in collaboration with youth and their caregiver(s), identify needs and goals, update regularly	[1, 20, 36, 41, 49, 50, 54, 56, 58–61, 64, 70, 75, 80, 81, 85, 88, 96, 97, 100, 103, 104, 106, 107]
	(3.2) Educate youth and their caregiver(s) about differences in CAMHS and AMHS programs and services	[13, 36, 39–43, 60, 62, 63, 65, 69, 74–77, 80, 85, 97, 100, 101, 105, 107]
	(3.3) Develop individualized transition plan in collaboration with youth and their caregiver(s) at least 6 months before planned transition	[1, 3, 35, 36, 41, 45, 47, 50–54, 56–59, 62, 63, 68, 75, 76, 79, 80, 83, 87, 90, 96, 100, 102–104, 108]
(4.0) Transition planning	(4.1) Identify all stakeholders involved in the transition	[59, 63, 75, 78, 79, 104]
	(4.2) In collaboration with youth and their caregiver(s), identify a provider (AMHS)	[38, 47, 56, 58, 59, 67, 102]
	(4.3) Confirm the AMHS agency/service eligibility criteria	[58, 64, 76, 94, 104]
	(4.4) Discuss optimal timing of transfer with youth and caregiver(s)	[45, 59, 60, 68, 76]
	(4.5) In collaboration with youth and their caregiver(s), complete and regularly update the individualized transition plan (including, for example: readiness assessment findings, goals and prioritized actions, medical summary, emergency care plan)	[13, 43, 65, 76, 105, 107]
	(4.6) Identify the most responsible clinician (i.e. transition/key worker) to ensure continuity in relationship and contact person during the transfer of care	[3, 4, 17, 35, 38, 40–44, 52, 55–60, 62, 63, 66, 67, 69, 70, 73–76, 78, 81, 83, 90, 93, 101, 104, 105, 108]
	(4.7) At least 6 months prior to transfer of care CAMHS clinician initiate transition planning with the AMHS provider, including holding joint working meetings or a period of parallel care; include youth and their caregiver(s) in meetings	[13, 14, 17, 20, 34, 35, 42, 44–46, 51, 52, 54, 56–60, 62, 63, 65, 67, 69, 70, 72, 75, 76, 85, 96, 98, 100, 103, 108]
	(4.8) Develop communication processes with primary care provider (i.e. family physician, nurse practitioner) to ensure they have consistent up-to-date medication and treatment information	[36, 44, 47, 52, 54, 56–58, 61, 66, 71, 75, 80, 85, 86, 88, 96, 97, 105]
	(4.9) Provide youth and caregiver(s) with contact information for self-care management resources, culturally appropriate community supports, and community mental health resources	[3, 47, 54, 56, 57, 59, 62, 63, 75, 80, 81, 88, 102, 108]
	(4.10) Provide community and health resources to the youth and their caregivers, in the event of withdrawal from AMHS service	[39, 52, 56, 60, 62, 67, 71, 73, 75, 77, 86]
(5.0) Transfer of care	(5.1) A specific meeting or case conference should be held with all stakeholders (i.e. youth, caregivers, CAMHS and AMHS clinician) to handover care	[34, 35, 41, 44, 56, 60, 65, 76, 80, 103]
	(5.2) Transfer young adult when his/her condition is stable	[13, 43, 50, 51, 56, 59, 65, 76]
	(5.3) Complete all documents in transfer package (e.g. referral letter, individualized transition plan, medical records), send to adult practice/external agencies, and confirm receipt	[13, 14, 35, 50, 54, 56, 57, 60, 61, 65, 76, 85]
(6.0) Transfer completion	(6.1) Most responsible clinician contact the youth and caregiver(s) 3–6 months after last CAMHS visit to confirm transfer to AMHS and offer consultation assistance	[34, 50, 54, 56, 57, 59, 75, 76, 99]

### Core element 1: transition policy

Transition policies discussed in the literature address age, service, and geographic boundaries. The components identified in this section describe how organizations and clinical programs ought to enhance their organizational readiness for transitions of their clients. This could be done through development and implementation of distinct policies, protocols, and staff training on the transition from CAMHS to AMHS. This review identified several documents that relate to policy and organizational issues and that examine the role of organizational readiness in the child to adult mental health service transition process (see Table 1). Hoffman et al. [1] state that youth, families and health care providers in the mental health system are governed by the policies that influence the clinical operation of programs and services, such as policies that dictate age and geographic boundaries. They argue that new policies are needed to enhance collaboration and ensure cross-boundary services are working together and pooling resources to provide optimal support for providers and families. These policies should be developed with an understanding of how clinicians and organizations operate programs and services, as well as how they will affect service users (i.e. youth and their caregivers) [51]. As such, an important aspect of developing transition policy is the inclusion of youth and their caregivers in its development [1, 3, 13, 35, 46, 52–60].

Recommendations were made about specific actions organizations can implement to facilitate successful transitions, including ensuring tailored education and staff training about transitions that is coordinated across both CAMHS and AMHS [1, 3, 4, 13, 20, 34, 35, 38, 39, 42–44, 49, 54, 57, 60–80]. This would include education on the unique needs of transition-aged youth [44, 68–70, 74]-specific diagnoses such as ADHD [42, 80], services in CAMHS, AMHS and the voluntary sector [67, 75, 77], and shared values and practices across CAMHS and AMHS [43, 69, 71, 75]. Several documents recommended that specific roles and responsibilities of all individuals involved in the transition process need to be determined and agreed upon [3, 13, 20, 34, 35, 56, 59, 60, 71, 76, 81–83].

The use of integrated care pathways (ICPs) was also suggested as a way to provide a clear overview of the organization’s approach to the transition process [1, 3, 20, 44, 46, 48, 51–53, 57, 59–61, 66, 67, 71, 73, 76, 80, 84–91]. ICPs are “structured, multidisciplinary care plans which detail essential steps in the care of patients with a specific clinical problem” [92]. They can increase consistency between programs and services, and formalize a currently informal process [49, 73]. An integral part of the ICP is a transition protocol that includes specific standards for communication, information sharing and record-keeping between CAMHS and AMHS [1, 3, 5, 13, 35, 42, 44, 48, 51, 52, 55–58, 60, 62, 63, 66, 67, 70–76, 79, 80, 85, 86, 89, 91, 93–100], as well as alternative planning for youth who are not able to connect with AMHS upon discharge from CAMHS or who withdraw temporarily from services [54, 56, 60, 75, 88].

Twenty-six articles identified the importance of using developmental readiness as a catalyst for transition rather than a chronological age (e.g. age 18) [4, 5, 13, 26, 40, 44, 45, 50, 52, 56, 59, 60, 62, 63, 68, 73–76, 80, 81, 83, 89, 90, 98, 101]. For example, Reale et al. [5] stated that developmentally defined boundaries rather than boundaries based on a fixed age will facilitate transition as a process rather than an event in time. The need for policies related to services and programs emphasized that care should be provided in accordance with the developmental needs of the youth. Establishing what is developmentally appropriate for youth ensures that their needs are being met and that transition is not simply an administrative process [48] prompted by age milestones [36, 48, 68, 102].

Last, 16 documents acknowledged the significance of assessing the quality of transition programs through a formal evaluation [34, 52, 54, 56, 59, 62, 63, 69–71, 75, 76, 79, 90, 98, 103]. However, no documents provided specific process or outcome indicators by which this could be achieved.

### Core element 2: tracking and monitoring

Twelve papers described the need for criteria to assist clinicians and administrators in identifying youth who will be transitioning out of CAMHS prior to the confirmed transition date or deadline [26, 52, 54, 63, 64, 76, 78, 80, 85, 103–105]. Youth frequently transition between services and out of care. As a result, there should be a formal process for tracking and managing these transitions. McManus et al. recommend developing specific transition criteria and a process to track and monitor individuals at all stages of the transition [106]. Six sources recommended that this process be facilitated by a flow sheet or log book [50, 54, 61, 76, 104, 106]. Additionally, the Care Quality Commission [105] of the UK recommended monitoring as an effective way to address wait times and to ensure that there is a standard practice for handling waitlists.

### Core element 3: transition readiness

Transition readiness assessments can be used to identify and review the transition needs and goals of youth and their families [20, 36, 41, 49, 50, 54, 56, 58, 59, 64, 70, 75, 80, 81, 85, 88, 96, 97, 100, 103, 104, 106, 107]. The readiness assessment should review diverse aspects of the youth’s life such as physical health, mental health, vocational, housing and educational needs [59, 103] as well as the youth’s own assessment of their strengths, skills and readiness for transition [56, 75]. In addition to the transition assessment, an individualized transition plan should be completed in collaboration with youth and their families, rather than by health care professionals alone [1, 3, 26, 36, 41, 47, 52, 54, 56–59, 62, 63, 68, 76, 79, 83, 87, 90, 102–104, 108].

The collaborative process of developing goals together with the youth and regularly updating the goals to align with the plan of care was identified as another meaningful contribution to transition [1, 50, 54, 56, 57, 60, 64, 76, 97]. The development and tailoring of the transition plan to youths’ unique needs requires time, with many documents suggesting a minimum of 6 months prior to transition [35, 45, 47, 50–54, 56, 63, 75, 76, 80, 96, 100, 102, 108]. Last, providing youth and their caregiver(s) with information about the differences between the CAMHS and AMHS is another component of transition readiness and was identified in 19 articles [13, 36, 39–42, 60, 62, 63, 69, 74–77, 80, 85, 97, 100, 101]. As noted by Swift et al. [77] education and information sharing may help set expectations and facilitate a feeling of preparedness among youth and their families.

### Core element 4: transition planning

Transition planning was identified as a core element with several associated components. The need to identify all stakeholders from CAMHS, AMHS, pediatric care, the education system and caregivers who will have a role in the transition was identified in several documents [59, 63, 75, 78, 79, 104]. Role clarity for all stakeholders is important, particularly when determining which agency takes primary clinical responsibility during the transition process [41, 53, 58, 67, 76]. Youth and their caregiver(s), and the CAMHS provider should collaborate in identifying an AMHS provider who will best suit their needs and transition goals [38, 47, 56, 58, 59, 67, 102]. The adult care provider will be responsible for supporting the youth after the transition to ensure their needs are being met [47]. This differs from a transition worker or “key worker”, who provides continuity throughout the transition process and transfer of care, following the youth as they move from child to adult mental health services. The presence of a key worker was identified as a core component of CAMHS–AMHS transitions in numerous documents [3, 4, 17, 20, 35, 38, 40–44, 52, 55–60, 62, 63, 66, 67, 69, 70, 73–76, 78, 81, 83, 90, 93, 101, 104, 105, 108].

Several documents recommended that a CAMHS provider initiate transition-related collaboration with an AMHS provider a minimum of 6 months prior to the transfer of care [44, 51, 58, 59, 65, 76, 103]. This collaboration could also be the catalyst for a period of parallel care between both agencies [13, 34, 44, 65, 69, 70, 75, 76]. Five documents identify the significance of transitioning at a time that is most appropriate for the youth [45, 59, 60, 68, 76]. The youth, their caregiver(s) and the CAMHS provider should agree upon the duration and time of the formal transfer. This might mean extending the period of time in CAMHS if required [68].

It is critical that the young person and their caregiver(s) are engaged in developing and regularly updating the transition plan [1, 34, 53, 54, 56, 59, 60, 70, 75, 79, 85]. This may include ensuring youth and their families are aware of the services available, know how they can prepare, what it means to transition, and what the transition process will entail [34, 41, 45]. As part of the transition process, youth should be provided resources and tools on strategies to manage their health. Publications recommended providing supports for self-management [3, 54, 56, 57, 59, 62, 63, 75, 80, 81, 102] as well as contacts for voluntary and community support services [47, 54, 56, 75, 108].

Primary care practitioners, such as a youth’s general practitioner (GP or family physician), are critical components of transition planning. Eighteen studies highlighted the importance of ensuring the youth’s GP is included in transition and care planning, especially when medications are involved [36, 44, 47, 52, 54, 56–58, 61, 66, 71, 75, 80, 85, 86, 88, 96, 105].

Five documents [58, 64, 76, 94, 104] identified the need to review the eligibility of the AMHS program or service in advance of transition to avoid discontinuity of care should the youth not meet the program or service’s criteria. It was suggested in multiple publications that health care providers in both CAMHS and AMHS should take responsibility for ensuring that the youth and their caregivers are made aware of the available mental health and community resources in the event of service gaps, withdrawal from AMHS or if additional support is required beyond the scope of AMHS [39, 52, 56, 60, 62, 67, 71, 73, 75, 77, 86].

### Core element 5: transfer of care

Transfer of care involves the logistical components of the discharge and intake processes during the transition [14, 35]. This includes the recommendation that the transfer from CAMHS to AMHS should only occur when the young person is stable [13, 43, 50, 51, 56, 59, 65, 76]. It is recommended that a formal handover of care should occur prior to transfer, include all stakeholders and be made during a multidisciplinary case meeting [34, 35, 41, 44, 56, 60, 65, 76, 80, 103]. A transfer package should be prepared ahead of this meeting and each stakeholder is given a copy of the package containing the dates of all future appointments, plan of care, goals, pending actions, and legal documents [13, 14, 35, 50, 54, 56, 57, 60, 61, 65, 76, 85].

### Core element 6: transfer completion

Transfer completion includes a component that speaks to what needs to occur after the youth has left CAMHS and completed the transfer of care. Several documents recommended that the CAMHS clinicians contact the AMHS service 3–6 months after the last CAMHS visit to confirm the youth has successfully engaged with AMHS and ensure there are no further questions about the referral or transition package [34, 50, 54, 56, 57, 59, 75, 76, 99].

## Discussion

Our review of the literature identified 56 articles and 30 gray literature eligible documents in an effort to: (1) define CAMHS–AMHS transition success; and (2) identify core components and corresponding transitions indicators that are being applied within the field. Two definitions of child-to-adult transition success were dominant among the reviewed documents. One was not specific to mental health [15] while the other was exclusive to CAMHS–AMHS transitions [76]. The latter was based on a recommendation of four criteria hypothesized to lead to CAMHS–AMHS transition success. This definition, however, did not include information on the implementation and measurement of these criteria, which raises issues about variability on how they are interpreted and applied. Although there is a lack of empirical evidence on the use of these criteria, the Singh [76] definition is widely applied in the CAMHS–AMHS transition literature. This lack of empirical evidence reflects the scarcity of intervention studies within the field, a conclusion supported by the systematic review from Embrett et al. [20] of the effectiveness of services and programs for youth transitioning from CAMHS to AMHS.

Our review delineated common core elements of transitions that multiple countries, organizations and disciplines agree upon. These core elements align with those of physical healthcare transitions [12], but have components that are unique to mental healthcare transitions. For example, the Got Transitions transition policy includes several components that emphasize the value of a shared view of how transitions should proceed with input from clinicians, youth, and their families [12]. The mental health literature takes this concept further, emphasizing that youth and their caregiver(s) should be included in decision-making at all phases of transition. Engaging in a shared planning process can promote common understanding between those who provide care (service providers and sectors) and those who receive care (youth and their families) [1, 51, 80].

Several core components of a successful transition were identified in this scoping review that was specific to the roles and responsibilities of the organization and service provider. However, it is important to acknowledge the broader context of system-level issues in mental healthcare systems that may constrain their effective implementation. CAMHS and AMHS are two separate systems with unique funding structures, service philosophies and target populations [1, 37, 58, 109]. Government funding allocations often dictate rigid age cut-offs, where service with CAMHS often ends at age 18 regardless of whether the youth is developmentally ready or connected to AMHS [1, 5, 72, 104]. This can result in service gaps while youth are placed on a waitlist for AMHS. Youth may also experience an eligibility criteria mismatch, where they find that upon transition out of CAMHS they are ineligible for service with AMHS because they do not meet diagnostic criteria [1, 4]. These issues have significant implications for the feasibility of the implementation of the core components. The close communication between CAMHS and AMHS that would be required to implement these core components would be severely constrained by service gaps and wait times. Even in an ideal scenario where AMHS is prepared to accept a young person directly following their transfer out of CAMHS, separate funding streams complicate the logistics of how providers would bill for collaborative efforts such as joint working meetings, case conferences, or parallel care [13, 104]. There is considerable debate within the literature about how to address these systemic problems, with options suggested including permitting service continuation from CAMHS based on developmental readiness, specialized services for 16–24-year-olds, expansion of AMHS eligibility criteria, and extending age cut-off for CAMHS to 25; however, no consensus has been reached [4, 67, 68, 83, 102, 104].

At the organizational level, the core components require further detail and clarity to be effectively operationalized. The literature does not clarify or suggest who should take ultimate responsibility for the enactment of the core components. Core components in core elements 1 and 2, for example, the development of policies, protocols and tracking mechanisms, suggest the involvement of organizational leadership. Meanwhile, the other core components implicitly point at clinicians. While collaboration with AMHS is encouraged, CAMHS clinicians seem to bear primary responsibility for the core components of transition readiness, transition planning, transfer of care and transfer completion. This raises the question of whether and how tasks such as conducting readiness assessments, developing transition plans, and compiling community resources can be integrated into clinicians’ existing workload in a manageable way, or if they require additional funding and resources [13]. Another key question centers around the appropriate involvement of family members and caregivers in the transition process. Although collaboration with family members and caregivers is interwoven throughout the core components, late adolescence is acknowledged as a period where youth move towards increased independence and autonomy from family [3, 53, 54]. The literature stresses that collaboration with youth and their family members needs to be negotiated with sensitivity [3, 13, 81], particularly in light of youth’s legal rights to confidentiality and increasing responsibility for self-management of their condition [53, 91].

Another important finding of this scoping review is the significant lack of measurable indicators and evaluative tools needed to assess the effectiveness of core components of transitions. Quality and process indicators are critical to the ongoing assessment, monitoring and improvement of mental health care. Evidence-based indicators also permit common reporting of performance measures across hospital and community mental health settings, and the identification of possible causes of discontinuities in care [110]. As such, future research should focus on the development, testing, and refinement of quality and process indicators to assess the performance of hospitals and community agencies in the transitions of youth between mental health services.

Despite the lack of indicators, several documents recommended a post-transition evaluation. However, none offered a framework or tool that would successfully achieve this and implementing this evaluation may be impeded by the lack of transition interventions and critical details on how to operationalize many of the core components. For example, several documents discuss the need for a key worker, yet there is little information about how to identify that individual, how to implement the role, and how to evaluate the role. A further example is the literature that recommends only transitioning the youth from CAMHS to AMHS when their condition is stable. However, there is little in the way of description about what ‘stable’ or ‘stability’ means for youth with mental illness and whether stability is realistic or necessary for the transfer of care. These two examples from this review illustrate the need to move beyond identifying and discussing youth and caregiver(s) needs for CAMHS to AMHS transitions to instead conducting research on the development, implementation and evaluation of promising new approaches to improve quality of transitions, and youth and caregiver(s) outcomes.

While our scoping review summarizes current knowledge about the core components of CAMHS–AMHS transition programs and services, it more importantly points to the need for empirical research on the impact and experiences associated with CAMHS–AMHS transitions. Conducting such research will require the development, implementation and evaluation of the quality and process indicators derived from the 26 core components identified in this review.

## Future clinical research directions

Moving forward, consensus on the importance and feasibility of the 26 core components identified in this scoping review is required. Employing a structured Delphi Study [111, 112] approach that includes youth, their caregivers, and clinicians can be used to achieve consensus. Such an exercise can provide the evidence needed to select core components and design interventions around them that will facilitate transition success for youth. To our knowledge, no Delphi or group consensus studies have been conducted with experts, including youth, parents, clinicians and administrators, to investigate which transition program components both enhance the transition experience and ensure continuity of care.

Another future clinical and research opportunity involves the development and evaluation of integrated clinical pathway (ICPs) [90]. These ICPs can be informed by the core elements and components identified in this scoping review and co-created in collaboration with youth, families and CAMHS and AMHS providers. ICPs tell the multidisciplinary team, the youth, and their caregiver(s) the steps to be undertaken to facilitate effective transitions and what to expect when youth transition [92, 113, 114]. Importantly, ICPs can provide services (including clinicians) with the information needed to evaluate planned care against what was actually delivered [92], and thus determine the effectiveness of a transition intervention or program. One recent publication, the National Institute for Health and Care Excellence (NICE) guideline “Transition from children’s to adults’ services for young people using health or social care services”, provides practical recommendations to improve the planning and quality of health care transitions. Although it is not specific to mental health care, it offers a model for programs aiming to improve the transition process and experience [56].

Another important focus for future research is the examination of funding structures and fees to understand how they can influence the CAMHS–AMHS transition process. There needs to be a thorough investigation of how mental health care funding and insurance programs at all levels of the mental health care system can impact on the success of transitions between mental health care services.

As this review focused specifically on CAMHS–AMHS transitions, we excluded any studies that describe the experiences of youth who do not transition directly into a service or program at AMHS (i.e. youth who return to their GP’s for mental health care). There is a need to better understand the needs of this group of youth—including their preferences for ongoing care. There is also a need to pay more attention to the roles and responsibilities of other resources and services, such as GP’s, post-secondary education institutions, and community mental health services, among others. The responsibilities of these organizations and services are vaguely defined at present, and it is unclear how they could be (or if they should be) incorporated into the transition process. There is also a need to clearly define the role of models of care that are focused specifically on providing mental health care to transitional aged youth (ages 15–24) in the CAMHS–AMHS transition process need to be clearly defined. This could be achieved by examining the needs of youth during this developmental period, and whether models of care promote continuity of care or provide another transition point that young adults and their caregivers must learn to navigate.

## Limitations

This scoping review has several limitations. First, the literature search may have unintentionally excluded some relevant documents, as it was limited to English language articles and documents. The use of English language only papers may have precluded the understanding of the depth of CAMHS to AMHS in a global context. The majority of the documents reviewed were from North American and European countries which may have limited our understanding of the nature of the issues in other countries. Next, while the search terms “transition to adult care” and “continuity of patient care” are Medical Subject Heading (MeSH) terms, ‘transfer’ and ‘transition’ are not. Last, the addition of these MeSH terms is recent; for example “transition to adult care” was added in 2011. In an effort to minimize the effect of these limitations, we screened the reference lists of all included articles to ensure that our literature search strategy did not miss any seminal documents. Last, this review focuses solely on typically developing youth with a primary diagnosis of mental illness. Future studies that focus on transitions among youth with neurodevelopmental conditions (i.e. autism) as a primary diagnosis should be conducted. This population might require specialty services outside the scope of mental illness that could lead to unique transition challenges. It merits a review solely based on those conditions.

## Conclusion

Our scoping review and the associated comprehensive list of core elements and components of CAMHS–AMHS transitions clearly demonstrate that it is possible to develop an integrated pathway and care coordination to improve transition experiences and outcomes. Such improvements would include better quality care, and care pathways that youth themselves have participated in developing, thus reducing the likelihood of youth ‘falling through the cracks’ as they move from child to adult mental health care. We also promote the engagement of youth and families as central decision-makers, an essential element of successful transitions in mental health care. Our scoping review clearly shows that there are important concepts and measures that can act as catalysts for change in current transition practices.
